# Infertility and childlessness: a qualitative study of the experiences of infertile couples in Northern Ghana

**DOI:** 10.1186/1471-2393-13-72

**Published:** 2013-03-21

**Authors:** Philip Teg-Nefaah Tabong, Philip Baba Adongo

**Affiliations:** 1 Ghana Health Service, Brong Ahafo Regional Hospital, Sunyani, Ghana; 2 Department of Social and Behavioural Sciences, School of Public Health, College of Health Sciences, University of Ghana, Legon, Accra, Ghana

## Abstract

**Background:**

Infertility is a global reproductive health issue that affects many individuals and couples. Despite the high prevalence of infertility in Ghana, no study has been done on the experiences of infertile couples in Northern Ghana. This study therefore explored the experiences of infertile couples in Northern Ghana using the Upper West Region as a case study.

**Methods:**

We interviewed fifteen childless couples, forty-five couples with children, and eight key informants using a semi-structured interview guide. We also carried out three focus group discussions; one for childless women, one for women with children and one for men with children. The data were transcribed, coded, arranged and analyzed for categories and themes.

**Results:**

Infertile couples are socially stigmatised and excluded from leadership roles in their communities. Couples without children are denied membership in the ancestral world thereby losing the opportunity to live again. Both males and females are engaged in sex with multiple partners to prove their fertility.

**Conclusions:**

Both men and women suffer from the social effects of childlessness. The desire to have biological children in a pronatalist society results in unhealthy practices. Health policy makers and gender advocates should be more concerned about infertility.

## Background

Infertility has been defined as failure to conceive after one year of regular unprotected sexual intercourse in the absence of known reproductive pathology [1]. However, epidemiological studies have revealed that in a normal population of heterosexually active women who are not using birth control, 25% will become pregnant in the first month, 63% within 6 months, and 80% within one year. By the end of a second year, 85% to 90% will have conceived [2]. Because some couples, who are not infertile, may not be able to conceive within the first year of unprotected sex, the World Health Organisation therefore recommends the epidemiological definition of infertility, which is the inability to conceive within two years of exposure to pregnancy [1]. Infertility may be primary or secondary. Primary infertility refers to infertility of a woman who has never conceived and secondary infertility refers to infertility of a woman who has conceived at least once before. The use of the ability of the female to conceive as a measure to differentiate between primary and secondary infertility is however problematic as it places responsibility for a couple’s infertility on the doorsteps of the female partner.

Worldwide, more than 70 million couples suffer from infertility, the majority being residents of developing countries [3]. Developing countries experience negative consequences of childlessness to a greater degree when compared with Western societies [4]. Regardless of the medical cause of infertility, women receive the major blame for the reproductive setback and they suffer personal grief and frustration, social stigma, ostracism and serious economic deprivation [5]. In Cameroon, infertility is a reason for divorce among the *Bangangte* tribe, causing a woman to lose her access to land distributed by her husband [6]. In Egypt, women go through a complicated ritual known as *kabsa* (a form of fertility-producing ritual) in efforts to overcome infertility [7]. Among the *Ekiti* of Southwestern Nigeria, infertile women are treated as outcast, after they die, and their bodies are buried on the outskirts of the town with those of people experiencing mental ill-health [8].

In view of the importance attached to parenthood in Africa, it is not surprising that infertility is reported to be considered a major cause for divorce and marital instability [9,10]. Consequently, infertile women commonly fear abandonment, divorce and polygamy [5,11]. In Northern Ghana, it is customary for the families of both the bride and groom to expect the announcement of an expected baby within a year of marriage and any delay in the signs of pregnancy by the woman is unacceptable [12]. The ability of the woman to give birth is generally viewed as a gain to the family of the woman’s in-laws for the bride wealth paid to the family of the woman.

There is a tendency of society to blame the woman for failed conception [13]. Consequently, the accepted norm is that infertility in a couple stigmatises the wife as barren and the husband as sterile. In this manner the implication of sterility presents men with an opportunity to abandon barren wives and de-stigmatise themselves by opting out of childless marriages. Participants’ in a study conducted in Cape Town indicated that they had to deal with being called *Idlolo*, meaning barren and *stjoekoe* (failure) [5]. Traditional customs, such as having to wear a scarf until a woman has a child also contributes to more pressure on women who suffer from infertility. In a study in South Africa, women expressed that they felt especially stigmatized and ridiculed in their families and in their communities. Participants’ described how they were sworn at, shouted at, cursed and victimized, seeing themselves as outcast, especially within their husbands’ families [5]. Prior to the realisation of involuntary childlessness, the individual probably identifies him or herself as a normal, conforming member of society [14]. It may be therefore, that social reaction to the disclosure of infertility plays a part in the establishment of a stigmatised identity. Women regard childlessness as discreditable, negative, and as representing failure. In addition, most experienced anxiety, isolation, and conflict as they privately explore the possibility of personal infertility. To avoid feelings of personal inadequacy, many women exclude themselves from gatherings such as baby showers or avoid their pregnant friends prior to revealing their involuntary childless status [14].

Studies have also revealed that the inability to have a child is often devastating to both partners; however, there are differences in men and women’s reactions to infertility. Prior research has tended to concentrate on the woman's experience while virtually ignoring the men [15]. Another study indicates that both sexes experience strong feelings of sorrow, isolation, urgency, guilt, and powerlessness [15]. Nevertheless, as a rule these feelings are generally expressed differently. In general, women are verbal and tend to seek out support during times of stress, while men use avoidance, minimisation, and denial. Contrarily, infertility has some positive effects in marriage such as bringing partners closer in the search for a solution to their problem. In a longitudinal cohort study of 2, 250 people who started fertility treatment, 25.9% of women and 21.1% of men were reported to have benefited [16]. Another study found that Muslim participants disclosed that they were afraid their husband might take a second wife. This is allowed by their religion so long as the first wife gives her blessing. However, this blessing is not required from a woman who cannot conceive [5]. In conclusion, women seem to submit to what they perceive as the consequence of infertility.

The treatment for infertility can either be traditional or biomedical. Traditional infertility services are common in Africa, and have been reported by many scholars and proponents of traditional medicine. Traditional health care is an important alternative source of understanding, coping, and managing health problems, including infertility, in the Gambia [17]. Medical therapy on the other hand is used to correct ovulation dysfunction (irregular or infrequent periods). If there are no underlying causes of ovulation problems (such as a thyroid disease), the first line of treatment is oral medication to induce regular menstrual cycles. For ovulatory dysfunction, representing almost 20% of female infertility [18], Clomiphene Citrate (CC) can initiate ovulation. Ovulation is induced in 50–70% of cases and, together with timed intercourse, the pregnancy rate varies between 15 and 25% per cycle with a low multiple pregnancy rate of 6–8% [18]. Surgery is sometimes required to treat conditions associated with infertility. The vast majority of surgical procedures used to address infertility can now be performed on an outpatient basis using a laparoscope (a type of endoscope) inserted through the navel and assisted reproduction technologies. For males, low sperm counts, deformed spermatozoa and inability to sustain an erection (impotence) are managed using medication. From the literature, there appears to be a gender bias in research on management of infertility as many studies have often focused on the women, reinforcing the belief that infertility is mainly caused by female factors.

Many of the previous studies have focused only on women, with little attention directed at the experiences of couples in a pronatalist society. It is in the light of this that this study was conducted to explore the experiences of childless couples in Northern Ghana. Such data are needed to complete the social science literature on this reproductive problem and to provide evidence-based data to guide the formulation of policies.

## Methods

### Ethics statement

Ethical approval for the study was received from the Ghana Health Service Ethics Committee. During the approval process for the study, the committee was explicit about the need to maintain confidentiality and anonymity whilst emphasising the need to obtain verbal or written consent from participants. In line with the approved procedure of obtaining consent for the study, verbal or written consent was obtained from participants. Verbal consent was obtained from participants who had difficulty reading the consent form and those who opted to give verbal consent. To those who gave verbal consent, the researchers read and translated the consent form into the preferred language of the participant. Participants were further asked to recommend a neutral member of the household to act as an independent witness in the consent process. A cover sheet containing the demographic information, except for the names and locations that were coded and kept separately, was used to document those who gave verbal consent. This procedure for obtaining verbal consent was approved by the Ethics Committee of the Ghana Health Service. To ensure confidentiality of participants who gave written consent, codes were used on the form instead of their names. The specific locations of the participants were also not reported as this could lead to easy identification of the couples who took part in the study. In addition, only codes were written on interview transcripts.

### Study area

The Upper West Regions is the smallest Region in Ghana with a population of 702,110 with 989 settlements. The region covers a total land area of 18,476 km^2^, with a population density of 32 persons per square kilometre [19]. The region is divided into eleven administrative districts and the people speak three main local languages namely: Wali, Dagaare or Sisali. The people of Upper West Region practice a patrilineal mode of inheritance; it is a typical patriarchal society with male dominance in decision-making. Polygamy is a common practice both by members of the Islamic community and those who profess African Traditional Religion. In all, there are sixty-five sub-districts, five district hospitals and a regional hospital in the regional capital. There is an orphanage located at Jirapa manned by the Sisters of Mary Immaculate, a Roman Catholic order of nuns.

### Data collection

In-depth interviews (IDIs), Focus Group Discussions (FGDs) and Key Informant Interviews (KIIs) were the main data collection methods used in the study. Four (4) trained data collectors were used in the study. These data collectors (Research Assistants) were put in two male–female groups. The male interviewers were assigned to male respondents and the female interviewers were assigned to female respondents with children. Feminist theorists argue that gender, class, and race affect all aspects of the research process, from the framing of the research question to the analytical approach [20]. Because in-depth interviews are social interactions [21], race, class, and gender inequalities are inherent in these interactions; interviewer/interviewee homogeneity was therefore adopted to overcome these challenges. The principal researcher carried out the interviews for both partners in couples with infertility. This was done because it was anticipated that some respondents might experience emotional distress narrating unpleasant past experiences. The principal researcher’s background in nursing and psychology therefore put him in a better position to detect promptly such emotional distresses and offer emotional support before referring such distressed respondents for counseling. This was done because qualified counselors were not readily available within the study area. The principal investigator used his experience in conducting interviews to overcome any challenge embedded in interviewer and interviewee heterogeneity and female respondents participated freely and actively. Interviews were terminated when any individual experienced emotional distress and were offered counselling and information they had provided was subsequently excluded from the analysis. Interviews were conducted in English or Dagaare depending on the language the respondent was comfortable with.

The interviews were tape-recorded with the permission of the participants; in addition, the interviewer took notes. In polygamous family, the male partner and the individual wives were interviewed starting from the senior of the wives in accordance with traditional norms of the communities. Younger wives in some instances were interviewed first upon the request of the senior wife in consultation with the husband. Codes were written on the interview guides and each recording was started by first mentioning the code on the interview guide to ensure data collected could be analysed as belonging to a couple for comparison to be made.

Field notes were written immediately after each interview. The field notes covered the initial interviewee reactions to the interview, including the first analytical reflections from the interview content, and any useful observations that could not be captured by digital recording. Notes were taken on the demeanor of the respondent, his or her body language and mood, and any informal conversation that took place before or after the interview. Each interview lasted between 30–45 minutes.

### Selection of participants

A snowball technique was used to recruit infertile couples into the study. The researchers first made informal contact with community health volunteers to interview them about their knowledge of couples experiencing infertility. The names and addresses of potential research participants were collected from the community health volunteers and such individuals were later contacted and interviewed. Infertile couples who were interviewed also gave the names of other couples with similar problems in the community. This approach was used until no new names of couples emerged. In light of the prevalence of polygamy in the community, the study considered a married union as the unit of analysis irrespective of the number of partners involved. Hence, fifteen (15) units of childless marriages were interviewed. However, three of the male partners without children in the study were married to two wives. This therefore increased the number of childless individuals in the study to thirty-three (33) comprising fifteen (15) males without children and eighteen (18) females without children. The male partners were aged between 35–63 years whilst the female partners were between 28–52 years of age. The duration of marriage among participant couples was between 3–25 years; couples adhered to Christianity (7 couples) or Muslim faiths (5 couples) or practiced African Traditional Religion (3 couples).

In addition to this, couples with children were also purposely selected and interviewed. In all, forty-five (45) households with children were recruited and interviewed. However, four of the males were married to two wives whilst one was married to three wives, increasing the number of women with children interviewed to fifty-one (51) with ninety-six (96) participants with children.

### Focus Group Discussions (FGDs)

Three Focus Group Discussions (FGDs) were organized. One was held with women in marriage unions without children whose male partners refused to take part in the group discussion. Their refusal to take part in the FGD was because infertility was widely perceived to be the inability of women to bear children and therefore a female issue. The researchers organized two (2) FGDs for male and female community members respectively, selected from rural and urban areas in which infertile couples were resident, to elicit normative ideas on infertility in the community. Members of groups were all married adults with children. Each discussion lasted for between sixty to ninety minutes. During FGDs, all participants were allowed to give their view on any subject raised before progressing to another theme.

### Key Informant Interviews (KIIs)

Eight (8) Key Informant Interviews (KIIs) were conducted with two gynaecologists who take care of infertile couples, an Islamic scholar who provided expert view on infertility and childlessness in the Islamic Community, a female Christian leader who provided support for childless women and two traditional medical practitioners who also provided care for infertile couples, the Head of the National Health Insurance Scheme and a manager of Private Insurance Company, both of the latter providing information on infertility related insurance policies in Ghana.

### Data analysis

The taped interviews were transcribed verbatim after repeatedly listening to the recordings and the resulting texts analysed by using thematic analysis. Repeated listening to tapes of interviews with participants is an essential, yet often neglected, area of analysis [22]. An attempt was first made to extract broad themes from the transcripts and then progress to identifying coded themes. In establishing themes, consideration was given to statements of meaning that were present in most of the relevant data. In an attempt to ensure the credibility of the findings, independent coders were used to verify or corroborate the themes extracted from the data. The data were analysed simultaneously with data collection. This allowed the researchers to progressively focus the interviews and observations, and to decide how to test the emerging conclusions. The transcripts were entered into QSR Nvivo 8© for analysis. The authors developed a codebook based on the major themes of the study. The major themes were transformed in tree nodes and free nodes. Based on the codebook the authors developed verified independently coded texts from the transcriptions. The emergent themes and sub-themes are discussed below, supported and illuminated by respondents’ quotes.

## Results

### Experiences of infertile couples

Infertile couples reported feeling depressed, and frustrated by the prescription of remedies by supposedly concerned individuals. Women generally reported more concern about their inability to give birth to a child than were men. Women without children in their old age are often branded as witches and abandoned by their relatives. Such women are not allowed to interact or take care of other people’s children as they are often accused of having “eaten up” all the children in their womb and could bewitch and cause the death of other people’s children.

“*Such women* (*infertile women*) *are dangerous to society in their old age because they become envious of other people’s children and will stand at nothing but cause the death of other people’s children*”- (An FGD female participant).

Men would have to battle with being branded as *Lankpolosoba* literally meaning “a man with rotten testes” or *Yokuusoba* meaning “a man with dead penis” The reasons for these descriptions were because the testicles are supposed to produce sperms and hence the inability of a man to impregnate the wife meant that the testes had lost their function. A strong penis was also required to convey the preformed babies (spermatozoa) to the woman; according to local cultural beliefs, spermatozoa are preformed humans. A penis that could not perform this task could therefore be described as dead. Such labels result in social isolation of men who are childless for fear of being openly insulted and disgraced.

“*I stay away from community gatherings except church services because people will point fingers at me and call me Lankpolosoba or Yokuusoba but neither are my testes rotten nor is my penis dead as am able to satisfy my wife; it is rather my wife who cannot conceive*”- ( A 56-year old infertile man in IDI).

There are however no such local terminologies to describe women. Many of the women mentioned their difficulty in witnessing or hearing children being abused by their parents. They confessed that they have great difficulty reconciling why they, women who so desperately wanted children and have unconditional love to offer, were being denied the chance to produce and parent a child. Participants reflected on the unfairness of their situation as they have the means or the desire to have a child, but are denied this prerogative, whilst other women who, in their opinion should not have children are fortunate enough to be blessed with children. They freely admit being envious, but also sometimes to being filled with anger especially when hearing or seeing a child being mistreated.

“ *I weep any time my neighbour beats her children and they are crying because this reminds me of my childlessness despite my unconditional love for children*”- (A 41-year infertile woman in IDI).

“*Hmm the people in my community are real gossips. They talk too much*. *They gossip a lot about it. Sometimes I pass by some places and all that I see is people pointing fingers at me*”- (A 36-year-old infertile woman in FGD).

### Experience of infertile couples within the family

Participants in this study reported unhappiness in their marriage as their ultimate dream of marriage is to have children. Their unhappiness also had a direct impact on their sexual life as many reported a reduced interest in sexual activity with their partners. Males especially reported that having sex was both for pleasure and for procreation, although procreation was the driving force for sexual intercourse among couples. However, with consistent failure of attempts to have children, the desire to have sex diminishes.

“*I have not had sex with my wife for more than six months now because it is a waste of energy*… *why do you continue to have fruitless sex with your wife. Akpetseshie* (*local gin*) *is now my wife; you take it to forget about the frustration you face in this family because of your childlessness*”- (A 52-year old childless man in IDI).

Males especially resorted to heavy drinking of alcohol or the local spirit known as *akpetseshie* to ward off their frustration. In a drunken state, sex with their partners was not a priority. Participants reported that a marriage without children is often perceived as a curse from God as the Bible states that children are a blessing from God, hence childlessness is a curse. Any marriage union without a child is therefore perceived as not sanctioned by God and is therefore a fertile ground for divorce or separation.

“*Any marriage without children is not ordained either by the gods of the land or God and hence such unions should not survive*”- (A male focus group participant).

There were mixed responses concerning the experiences of couples within the extended family setting. While some people reported support from their families, others blamed their families for their unhappiness. Women who were perceived to be responsible for the couple’s childlessness reported that they were badly treated by their mothers-in-law who are demanding grandchildren.

“*I live in perpetual fear as my mother in-law can come here at any time and start casting innuendoes against me for bewitching her son*”- (A 36-year infertile woman in IDI).

Other respondents reported having a good relationship with the extended family. Elite group of respondents reported more support from their families than uneducated couples. Elite couples were under less pressure to beget children because the families were reliant on them for financial support.

“*We are blessed with a supportive family who will not ask sensitive questions but just encourage us to do what makes me happy*”- ( A 43-year old infertile elite woman in IDI).

### The stigma of infertility in the community

Children are indicators of a man’s wealth and prosperity in the community. Men without children therefore do not receive the same respect as fathers. Some men reported that they were excluded from leadership roles in their communities because they did not have children. To be able to perform such leadership roles the infertile man will have to undertake certain rituals, after which a *Saabie* (undertaker for clan members) is allowed to impregnate the infertile man’s wife on the husband’s behalf. However, this does not take away the humiliation and stigma, as the biological fathers in such arrangements are not able to keep this contract secret. The biological fathers in such a partnership are also reported to abuse the women sexually in some instances.

“*I had to allow a Saabie* (*a male undertaker of my clan*) *to sleep with my wife to beget children for me because I was supposed to become the head of my family but I did not have children. This Saabie however has now made it a point to be sleeping with my wife*”- (A 63-year infertile man in IDI).

Other individuals determined to prove their fertility have intercourse with multiple partners, hoping either to become pregnant or to get a woman pregnant. Both males and females were reported to engage in sex with multiple partners to prove their fertility. However, extramarital sex is generally unacceptable and attracts serious social consequences especially for women. A woman caught engaging in extramarital sex has to go through some purification rituals before she can either cook for, or have sex with, her husband. Failure to go through these rituals is believed to result in a condition known as *moora*, which they believe has the same clinical manifestations as HIV/AIDS. This is because the bride price that is paid to the family of the woman confers on the man the exclusive sexual rights and any contrary behaviour attracts serious social consequences.

“*I had to go through rituals, including been stripped naked and thorns used to scour my private parts because I wanted to save my marriage by engaging in extramarital sex, in order to become pregnant and give birth to a child for my husband. Unfortunately, luck eluded me when I was caught and had to be purified*”- (A 38-year-old infertile woman in IDI).

Some childless men are compelled to acquire a second wife in an attempt to prove their fertility in an effort to escape the ridicule and stigma that goes with being childless in the community.

“*I had to marry my second wife because I lived with my first wife for 10 years without a child. One day I was passing a neighbour’s house, and saw pawpaws that he had put on display for the purpose of selling them. I asked if he was selling the pawpaws for me to buy some. My neighbour looked me in the face and told me that it was only an impotent man that sells pawpaw; implying that because I did not have a child I did not know that pawpaw was a fruit for children, and men with children save pawpaws to give to their children. This made me marry a second wife and we have lived for 10 years without a child too*”- (A 45-year-old infertile man in IDI).

The stigma experienced by infertile couples does not occur only when they are alive, it also continues after the person’s death. Couples without children are denied membership in the ancestral world. Rituals that confer membership in the ancestral world cannot be performed because it is the practice that the children of the deceased go to the bush to harvest a special stick known as the *kpiendaa* which is kept at the ancestral room after some rituals symbolizing that the individual has reached the ancestral world. This rite of passage is very important in the lives of all adults as it indicates the final transition of the individual to the ancestral world. The general belief is that there is another world after death and individuals who become ancestors will have the opportunity to live again. If this does not occur, it implies extinction of the family lineage and unrest for the soul of the deceased. Such souls are believed to wander about and will never reach their maker.

“*I have done whatever I can in this community; however, my name will disappear immediately I die and I can never be remembered nor be an ancestor, my name can never be mentioned in any ritual in this community, it is disheartening*”- (A 62-year infertile man in IDI).

Infertility also presents stressors in the financial domain. A couple may incur tremendous financial expenses in an attempt to stop at nothing until a pregnancy or a live birth is achieved. Respondents stated that fertility services were available as spiritual and herbal remedies were readily available in the community. However, the cost of treatment was a challenge as items often requested are not within the financial capability of the couples. The biomedical health facilities resourced for infertility are often at the district level and are limited in terms of assisted reproductive technologies. In addition, many of the procedures are not covered by the national health insurance scheme. A National Health Insurance Scheme Manager alluded to the fact that many of the treatments for infertility are not covered by the scheme and they have had reported instance of exploitation of couples even for procedures that were covered by the scheme. A check with private insurance companies also revealed that currently their policies do not cover infertility.

For example, a 37-year old respondent said in IDI:

“*I believe it is spiritual; I have been to so many places but did not get any results, some people have such problems* (*infertility*) *because they are being attacked by bad spirit. In the hospital, once they write infertility on your card, you are made to pay for every treatment including those covered by the national health insurance. It is like a curse to be diagnosed as suffering from infertility in Ghana*”.

The people of Northern Ghana believe in pronatalism to the extent that childlessness is highly stigmatised and this was a well-entrenched theme across all groups and interviews with community members. It is not simply the absence of children, which can create problems for couples. For a significant majority of participants, bearing no sons, or having only one child constituted a form of infertility where community norms dictate that large families are preferable and that sons are indispensable.

“*A couple without a male child is the same as not having children at all, after all your name will still become extinct as there is no male to name children with your surname*”- (A man with children in a focus group discussion).

Infertility is seen almost exclusively as a woman’s problem. All participants in the study agreed that women are blamed for childlessness. ‘*It’s always the woman’s fault’* was a very common theme. Many of the interviews with childless individuals and couples in the study also revealed negative experiences; unsupportive reactions to infertility from family members and painful social scrutiny. Several infertile women reported negative behaviour towards them from their partner’s family. Some of the women felt that childlessness denied them full membership of their husband’s family, and that their role in the family would not become secure until they had a child.

“*Even though I am married to the first born in this family, my contribution is valueless because I have no child. I only listen during family meetings because am afraid to be insulted by my colleague women*”- (A 52-year old infertile woman in IDI).

### Coping strategies

Participants indicated that there are days when the reality of life without children just seems too much to handle, but that they have found ways to deal with it. Participants reported that having a coping strategy was important in order for them to continue their lives without children. Methods of coping included depending on internal resources such as inner strength, self-confidence, and true acceptance of their fate, being able to rely on a support structure or trying to move on by focusing on the future.

“*Sometimes it is really difficult to imagine life without children, but you have to find a way of coping to prevent doing something stupid; this you do by redirecting your energy towards other ventures*”- (A participant in FGD with childless women).

Infertile couples narrated going through stages similar to the grieving process described by Kubler-Ross (1969): denial, anger, bargaining, depression and acceptance [23]. Many individuals went into marriage with a strong hope to become mothers and fathers. However, the possibility of infertility began to occur to them after several months of unprotected sexual intercourse with their partner without conception. Even where no official diagnosis was established, many reported becoming angry because of failed attempts to become pregnant or make their wives pregnant. Denial was a common theme and many reported mixed angry-denial mood or a fluctuation between anger and denial. Denial is facilitated by the assumption that, since their parents were fertile, they will also be able to beget children.

“*I told myself it was impossible for me to be infertile, several months after marriage and I have never missed my period*”*-* (A 41-year old infertile woman in FGD).

They subsequently begin to shop for solutions whilst bargaining with God or gods depending on their religious belief. Many couples reported that their initial reaction to the diagnosis of infertility is also denial. “*It cannot be true*” was a common phrase many infertile individuals reported having used when they were first told about their infertility status. Women reported coping very well with male partners who are clinically diagnosed as infertile. However, women who were perceived to be responsible for the childlessness of a couple live in perpetual fear of acquiring rivals.

“*Every new day comes with its challenges, you need to be so submissive to your husband to prevent getting a rival. I had to fight my mother in-law to prevent her from bringing me a rival*”- (A 39-year old childless woman in FGD).

Coping in the social context or in the community was avoidance of situations that may provide a reminder of their childlessness. Others take inspiration from the biblical story of Abraham and Sarah who had to wait until their old age before begetting a child (Genesis 21), hoping for a miracle to happen.

“*God can give you a child at any age as he did to Abraham and Sarah in the bible, so I am waiting for God’s time*”- (A 46-year old infertile man in IDI).

Female respondents reported aspiring for excellence in others areas of their lives as this was another way to earn respect in the community.

“*I had to go to university to do my first degree because I waited several years to become pregnant in vain, after which I proceeded to do a masters degree because you cannot lose in two areas. If you do not have children, get the degrees and you will still be respected*”- (A 45-year old infertile woman in IDI).

Infertile individuals adopted both healthy and unhealthy coping mechanisms. Acceptance of the situation and remaining faithful whilst praying to God for mercy was widely reported among Christians and some Moslems. Another healthy coping strategy that was also reported in both individual interviews and FGDs was redirecting their energy into economic ventures. This was another way to earn respect in society. However, many reported unhealthy coping strategies.

Social isolation, abuse of alcoholic beverages, and engaging in sex with multiple partners were widely reported among men. In an initial response before agreeing to be interviewed, a 48-year old childless man stated emotionally, “*young man, I have for years abused alcohol because of this situation* (*infertility*) *but I have forgotten about it and you have come to remind me about it again*”.

### Methodological considerations

The fact that the first author was a man and interviewed women as well as moderated the FGDs with women could have affected the results of this study. This according to feminist theorists can affect the responses of interviewees. However, the women seemed to have participated freely and actively. The women also indicated that they were more comfortable discussing their childless status with men than with women. Another limitation of the study was related to the translation of the interviews and FGDs into English for analysis. Though independent individuals were used to undertake the translation and the translations were verified, it is possible that some of the statements could have lost their original meanings. Some of the local terms used could not be directly translated to English. To help mitigate this problem, emphasis in analysis was placed on overarching ideas rather than specific word choices or phrasing.

## Discussion

The results of the study indicate that infertile couples are stigmatised in Northern Ghana. Both men and women are stigmatized with men being described in local terms such as *Lankpolosoba* (man with rotten testes) or *Yokuusoba* (man with a dead penis) in the Upper West Region. Similar findings were found in a study in South Africa where it was reported that infertile men are stigmatized, verbally abused and lose their social status in the community [24]. However, participants in a study conducted in Cape Town indicated that women had to deal with being called *Idlolo*, meaning barren and *stjoekoe* meaning failure [5]. Contrary to these findings, the present study did not reveal the use of such local terms to described infertile women. The study further revealed that women were frequently blamed for a couple’s infertility even when the aetiological factors had nothing to do with the woman. There is a tendency of society to blame the woman for failed conception [13].

Both men and women were reported to be denied leadership roles based on infertility; however, men appeared to be better placed to adapt to that as they could arrange with another man to impregnate their wife for them. This practice of allowing a different man to sleep with your wife to beget children on your behalf or having sex with multiple partners have implications for the prevention and control of Sexually Transmitted Infections (STIs) in Northern Ghana. The use of condoms is one of the key strategies advocated for the prevention of STIs. However, since the principal reason for having sex with multiple partners is to achieve a pregnancy, condom use is clearly impractical. This therefore has health implications, as this can lead to high incidence of cross infections with STIs if one of the partners should have an infection. Women who engage in sex with multiple partners are also more predisposed to cervical cancers. The greater the number of sexual partners a woman has without the use of condoms, the greater her risk of coming into contact with Human Papilloma Virus ( the virus that causes cervical cancer), and later developing cervical cancer [25].

The effects of infertility on inter-partner relationships varied. As some couples reported cordial relationships, other reported a decline in sexual activity. Those who reported marital instability were mostly engaged in excessive alcohol intake, which has untoward effects on their health. The abuse of alcohol, apart from predisposing the individuals to cancer of the stomach and cirrhosis of the liver, can also result in spousal abuse. Poverty is endemic in Northern Ghana and many women are not empowered. The findings of this study therefore indicate that partners and families may secretly be abusing women as a result of childlessness. Stripping a woman naked and scouring her private parts with thorns, as a ritual to purify a woman caught in extramarital affairs is one such example. Gender advocates should be concerned about the role of infertility in spousal abuse and efforts should be tailored towards empowering women in Northern Ghana.

Previous studies have revealed that infertility has some positive effects in marriage such as bringing partners closer together in the search for a solution to their problem [16]. In contrast, the present study did not reveal the search for remedies as a source of intimacy for couples, since many couples sought either individual treatments or the women were the one seeking treatment alone. Seeking remedies individually emanates from couples blaming one another for their inability to have children and this reinforces the practice of the partners engaging in extramarital affairs to demonstrate their fertility.

The study further revealed that childlessness affects both the physical and spiritual well-being of the individual. Infertility has inter-generational effects, as couples without children do not have the opportunity to live again. Reliving is achieved through either reincarnation or having the opportunity to live in an unseen world. Among the people of Kassena-Nankana in the Upper East Region in Northern Ghana, the unseen world is believed to harbour all departed souls and forms a spiritual home for ancestors [26].

Respondents reported both healthy and unhealthy coping strategies. Though previous studies have shown that women are expressive and therefore will talk about their infertility [15], this study generally revealed that among women in Northern Ghana, infertility is not usually discussed in public and infertile women would have to rely on internal coping strategies. Having faith in God and hoping for a miracle to occur was an essential coping mechanism that was employed by infertile couples. Some also redirected their energy to excel in other areas that are respected in society such as academic achievement. Similarly, women were reported to cope through drawing on their Christian faith [27]. Aspiring for higher achievements in their field was also a common coping strategy reported by women in the study. This coping strategy has positive impact in the lives of an infertile woman as it reduces the depression emanating from the stigma associated with infertility.

## Conclusions

It is clear from the study that although infertility is an important public health issue, it has received less attention from health policy makers in Sub-Saharan Africa. Infertile individuals endure severe stigma and social derision in silence. This stems from the fact that there are no appropriate social and medical structures within the formal health sector to deal with the challenge. The derision occurs both in the physical and spiritual world as childless adults are denied membership in the ancestral world after they die. To escape this ridicule, couples are compelled to engage in unhealthy sexual practices that could have serious consequences on their reproductive health. This calls for the need to prioritized and integrated health services relating to infertility into existing reproductive health strategies to reduce the burden of infertile individuals.

## Authors’ contributions

PT-NT designed the study, participated in data collection and analysis and prepared the draft of the manuscript; PBA provided scientific advices on the design of the study and data analysis. All authors read and approved the final manuscript.

## Pre-publication history

The pre-publication history for this paper can be accessed here:

http://www.biomedcentral.com/1471-2393/13/72/prepub
